# 2-Dimensional foot FE models for clinical application in gait analysis

**DOI:** 10.1186/1757-1146-7-S1-A73

**Published:** 2014-04-08

**Authors:** Alessandra Scarton, Annamaria Guiotto, Zimi Sawacha, Gabriella Guarneri, Angelo Avogaro, Claudio Cobelli

**Affiliations:** 1 Department of Information Engineering, University of Padova, Padova, 35131, Italy; 2 Department of Clinical Medicine and Metabolic Disease, University Polyclinic, Padova, 35131, Italy

## Background

Foot ulcerations are one of the most common and invalidating complications which affect the diabetic patients [1,2]. Several two-dimensional (2D) finite element (FE) models of the foot have been developed in the last decades in order to understand what are the causes and to decrease their progress [3-5].

The aim of this work was to create four 2D FE models of an healthy and of a diabetic neuropathic subject integrating kinematic, kinetic and pressure data and to validate them by means of a comparison between experimental and simulated pressure values. These models could represent a tool for clinical applications in order to prevent the development of the diabetic ulcers.

## Methods

Foot biomechanical analysis was carried out as in [6,7] on 10 healthy (age 58.7±10 years, BMI 24.5±2.6 kg/m^2^) and 10 diabetic subjects with neuropathy (age 63.2±6.4 years, BMI 24.3±2.9 kg/m^2^). The experimental setup included a 60 Hz 6 cameras stereophotogrammetric system (BTS S.r.l, Padova), 2 force plates (FP4060-10, Bertec Corporation, USA) and 2 plantar pressure systems (Imagortesi, Piacenza). The signals coming from all systems were synchronized as in [6,7].

**Figure 1:**
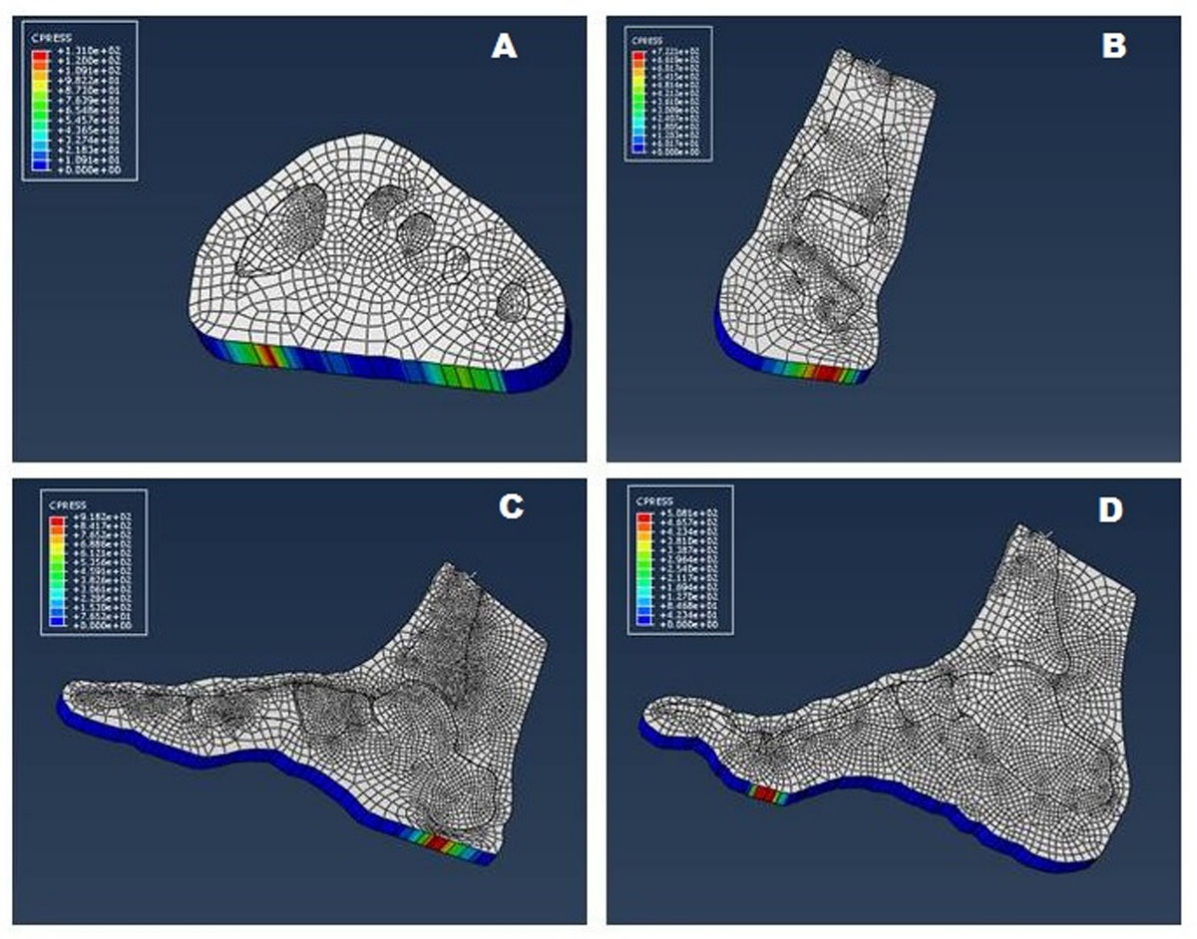
The figure shows the four models developed for the diabetic subject. A) 1^st^-5^th^ metatarsal head model; B) Through malleoli model; C) 1^st^ metatarsal -calcaneus model; D) 2^st^ metatarsal -calcaneus model.

Four 2D FE models of the foot were developed from MRI images of a healthy and a diabetic subject (Figure 1). The modeled section were chosen as typical areas of ulcers development and according to the position of the marker in the gait analysis protocol: the slice passing through the first and the fifth metatarsal heads, the slice passing through the malleoli, the slice passing through the calcaneus and the second metatarsal head and the slice passing through the calcaneus and the first metatarsal head.

The displacements of the markers determined from the gait analysis data for each patient in four instances of the stance phase of gait (initial contact, loading response, midstance and push-off) were used as input for the simulations. The validations of the models have been performed computing the RMSE between the experimental and the simulated plantar pressures in percentage of the experimental peak value.

## Results

Results for the diabetic subjects are shown in Table 1. No significant differences were found between the healthy subjects experimental and simulated pressures.

**Table 1:** RMSE between the experimental and the simulated plantar pressures in percentage of the experimental peak value, in four instances of the stance phase of gait and for the four models.

	Initial contact	Loading response	Midstance	Push-off
**1^st^ metatarsal -calcaneus model**	25.08	20.43	33.26	24.69

**2^st^ metatarsal -calcaneus model**	22.20	24.68	37.82	41.67

**1^st^-5^th^ metatarsal head model**	-	45.77	46.34	46.25

**Through malleoli model**	35.36	42.12	46.58	-

## Conclusions

Even under the restrictive assumptions of 2D representation, which is inadequate for a complete model of the complex mechanics of the foot, it is possible to run fast computational simulations that provide useful information for the clinicians towards a prevention of plantar ulcer formation.
